# Reannotation of the cultivated strawberry genome and establishment of a strawberry genome database

**DOI:** 10.1038/s41438-021-00476-4

**Published:** 2021-03-01

**Authors:** Tianjia Liu, Muzi Li, Zhongchi Liu, Xiaoyan Ai, Yongping Li

**Affiliations:** 1 Institute of Fruit and Tea, Hubei Academy of Agricultural Sciences/Fruit and Tea Subcenter of Hubei Innovation Center of Agricultural Science and Technology, Wuhan, China; 2 Department of Cell Biology and Molecular Genetics, University of Maryland College Park, College Park, MD USA; 3 School of Life Sciences and State Key Laboratory of Agrobiotechnology, Chinese University of Hong Kong, Shatin, Hong Kong China

**Keywords:** Non-model organisms, Transcriptomics

## Abstract

Cultivated strawberry (*Fragaria* × *ananassa*) is an important fruit crop species whose fruits are enjoyed by many worldwide. An octoploid of hybrid origin, the complex genome of this species was recently sequenced, serving as a key reference genome for cultivated strawberry and related species of the *Rosaceae* family. The current annotation of the *F. ananassa* genome mainly relies on ab initio predictions and, to a lesser extent, transcriptome data. Here, we present the structure and functional reannotation of the *F. ananassa* genome based on one PacBio full-length RNA library and ninety-two Illumina RNA-Seq libraries. This improved annotation of the *F. ananassa* genome, v1.0.a2, comprises a total of 108,447 gene models, with 97.85% complete BUSCOs. The models of 19,174 genes were modified, 360 new genes were identified, and 11,044 genes were found to have alternatively spliced isoforms. Additionally, we constructed a strawberry genome database (SGD) for strawberry gene homolog searching and annotation downloading. Finally, the transcriptome of the receptacles and achenes of *F. ananassa* at four developmental stages were reanalyzed and qualified, and the expression profiles of all the genes in this annotation are also provided. Together, this study provides an updated annotation of the *F. ananassa* genome, which will facilitate genomic analyses across the *Rosaceae* family and gene functional studies in cultivated strawberry.

## Introduction

Cultivated strawberry (*Fragaria* × *ananassa*), an octoploid species (8*n* = 56) of hybrid origin, is an economically important fruit crop species worldwide whose fruits are appreciated by many due to their attractive appearance, unique flavor, and health benefits. The earliest genome assembly of the *F. ananassa* variety Reikou was based on the Roche 454 and Illumina sequencing platforms and published in 2014^1^. At the time, the algorithms for heterozygous genome assembly were not well established, and as a result, the genome integrated the homoeologous sequences into a haploid genome, called FANhybrid_r1.2, which comprised 212,588 sequences with an N50 length of 5.1 kb. Recently, a near-complete chromosome-scale assembly of the cultivated strawberry (*F. ananassa*) genome was published^2^. This new and improved assembly has a contig N50 of approximately 79.9 kb and was assembled using Illumina, 10X Genomics, and PacBio long reads. This chromosome-scale genome that comprises the A, B, C, and D subgenomes provides an invaluable resource for marker-based breeding and genomic and functional analyses of cultivated strawberry.

In addition to genome assembly, high-quality genome annotations are essential during genome assembly and improve the utility of the genome. The advantage of Illumina technology has stimulated the construction of transcriptome resources for many Fragaria species, particularly the wild relative *Fragaria vesca*^3–5^. However, Illumina-based short RNA sequencing reads pose a major challenge in transcript assembly and annotation^6^. Compared with short-read sequencing, long-read sequencing produced via Oxford Nanopore Technologies (ONT) and Pacific BioSciences (PacBio) can provide full-length transcript sequences, which greatly improve the accuracy of gene structure annotation^5,7^. Furthermore, the ONT and PacBio technologies also benefit analysis of alternative splicing (AS), thus facilitating a more comprehensive gene expression analysis. Alternative splicing plays important roles in gene expression and function in strawberry and other plant species^8–11^. For instance, the polygalacturonase gene in strawberry (*FaPG*) produces an alternative transcript with a premature termination codon that affects fruit firmness^10^. Our previous study also showed that mRNAs with retained introns are inaccessible to ribosomes, and regulated removal of the retained intron may serve to quickly release the ‘stored’ mRNA for protein synthesis and organ growth^11^. For allopolyploids harboring large numbers of homoeologous genes, differential transcript splicing may provide additional insights into subgenome dominance and evolution of new traits. Recently, a high-quality octoploid strawberry reference transcriptome was established via SMRT sequencing technology^12^. This SMRT RNA sequencing resource provides opportunities to further improve *F. ananassa* genome annotations.

Previously, using a well-established annotation pipeline, we created two high-quality annotations of the genome of *F. vesca* (v2.0.a2 and v4.0.a2)^5,13^. The *F. vesca* genome is one of the ancestral genomes of *F. ananassa* and supposedly is the dominant subgenome in *F. ananassa*^2^. To improve the annotation of the *F. ananassa* genome, we utilized the pipeline with available RNA-seq datasets, including one PacBio full-length sequencing dataset and 92 RNA-seq libraries obtained from flower, petal, leaf, root, stem, and fruit (receptacle and achene) tissues at different developmental stages or under different treatments (Table S1)^2,12,14–18^. As a result, the new and improved annotation, named v1.0.a2, has 108,447 protein-coding genes, with 97.85% complete BUSCOs. We also constructed an SGD website (http://www.strawberryblast.ml:8080/strawberry/viroblast.php) for strawberry gene homolog searching and annotation downloading. Additionally, the expression profiles of all annotated genes in *F. ananassa* during achene and receptacle development are provided in this study. Overall, these new annotation and gene expression profiles will facilitate future gene function studies in strawberry.

## Results and discussion

### Reannotation of the *F. ananassa* genome via our prior pipeline

**Fig. 1:**
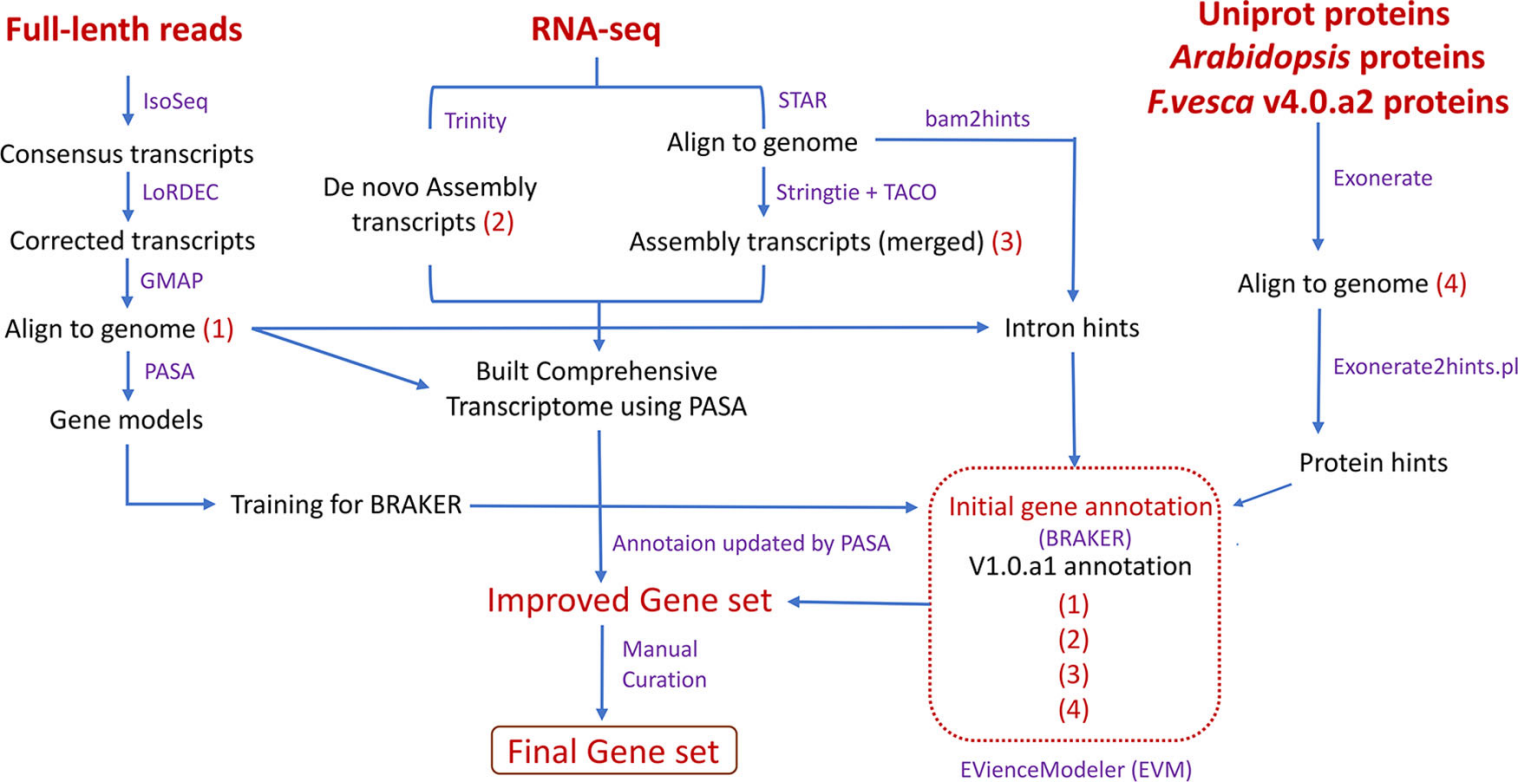
Annotation workflow for *F. ananassa* protein-coding genes. The best gene models generated from full-length transcripts were used for training for BRAKER. RNA-seq datasets were used to construct comprehensive transcriptomes via genome-guided and de novo assemblies. *F. vesca* v4.0.a2 annotation and UniProt and *Arabidopsis* protein sequences were also inputted into BRAKER. The red dotted box contains the input evidence for EVidenceModeler. In addition, manual curation was conducted to ensure the accuracy of the annotation

In this study, we created an updated annotation of the most recent *F. ananassa* genome (Edger et al.^2^). The new annotation is named version 1.0.a2 (v1.0.a2). The v1.0.a2 reannotation process incorporated full-length transcript data and extensive RNA-seq data from different tissues and at different developmental stages for AS isoform identification and gene structure updates (Fig. 1). First, we used BRAKER v2.1.5^19^ to generate an initial protein-coding gene annotation. The input data for BRAKER included the following: (i) BRAKER-trained models; (ii) intron hints converted from aligned full-length transcript sequences; (iii) intron hints converted from aligned RNA-seq reads and de novo transcripts; (iv) protein hints generated from mapping UniProt, *Arabidopsis* Araport11 and *F. vesca* v4.0.a2 protein sequences; and (v) repeat masked genome data. The SMRT full-length sequences were obtained from pooled strawberry tissues (including dwarf stem, flower, mature leaf, and fruit tissues at six different stages)^12^. Illumina RNA-Seq libraries were obtained from a series of different tissues of *F. ananassa*, including flower, petal, root, leaf, stem, and fruit tissues at different developmental stages or under different treatments (Table S1)^2,14–18^. EVidenceModeler (EVM) software^20^ was then used to combine the different types of evidence into consensus gene models, including gene models predicted by BRAKER, mapped full-length sequences, protein sequences, de novo sequences, and genome-guided transcripts (Fig. 1). Integrative Genomics Viewer (IGV)^21^ was used to inspect new annotations across the entire genome and select the optimal gene models by comparisons with the mapped RNA-seq reads; approximately 2000 (1.8%) genes were manually curated. Finally, we obtained the new annotation v1.0.a2, which contained a final set of 108,447 genes (Table S2).

**Fig. 2:**
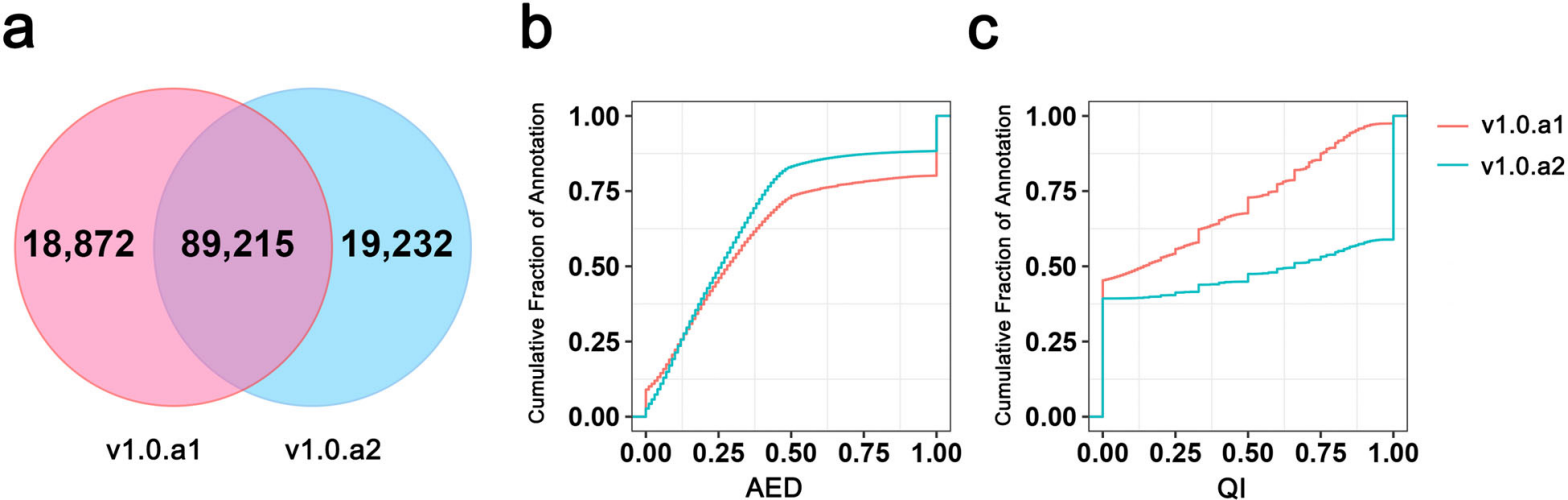
Comparison of v1.0.a1 and v1.0.a2 annotations. **a** Venn diagram showing the common and unique gene structure of the CDS region between v1.0.a1 and v1.0.a2. **b** Cumulative AED distribution for the v1.0.a1 and v1.0.a2 annotations. **c** Cumulative QI distribution for the v1.0.a1 and v1.0.a2 annotations

*F. ananassa* annotation v1.0.a2 contains 108,447 protein-coding genes and 127,701 transcripts (Table 1). The locus IDs and position for the newly modified genes in both versions are listed in Table S3. The gene models (coding DNA sequence regions) were compared between v1.0.a1 and v1.0.a2 in gtf format by Cuffcompare^22^. The transcripts with a class_code ‘=’ classified by Cuffcompare were defined as the same gene model. As a result, a total of 89,215 genes, 82.63% of those of v1.0.a1 and 82.27% of those of v1.0.a2, were shared between the two genome annotation versions (Fig. 2a). In the v1.0.a2 annotation, 360 novel genes (class_code ‘u’) were added (Table 1), and 6993 genes in v1.0.a1 were removed. Furthermore, the model of 19,174 genes was updated and modified (remaining class_codes). The gene IDs in the previous annotation (v1.0.a1) remained the same in the new v1.0.a2 genome, following the format FaxC_XgXXXXX. The statistics comparing v1.0.a1 and v1.0.a2 are shown in Table 1. A total of 86,622 genes were found to have 3′UTRs and/or 5′UTRs, representing approximately 79.9% of all the annotated genes. The average number of exons per gene increased from 5.4 to 5.7. The new annotation also contains alternatively spliced or alternatively initiated transcripts. A total of 30,298 transcripts from 11,044 genes were found, resulting in an average of 1.2 transcript isoforms per gene at the whole-genome scale. In addition, 17,265 genes were assigned GO terms in v1.0.a2, compared to 16,569 genes in v1.0.a1. A total of 76,925 genes acquired functional annotations in v1.0.a2, 3009 more than the number in v1.0.a1 (Table 1).

**Table 1:** Summary of the v1.0.a2 annotation.

Type	v1.0.a1	v1.0.a2
**Protein-coding genes**		
Number of genes	1,08,087	1,08,447
Mean length of genomic loci	3158	3015
Mean exon number	5.4	5.7
Mean CDS length	1443	1071
Mean length of introns	363	347
Genes with a 5′UTR	47,356	82,253
Genes with a 3′UTR	51,473	84,204
Genes with both a 5′UTR and 3′UTR	42,076	79,832
Mean 5′UTR length (bp)	324	428
Mean 3′UTR length (bp)	588	525
Number of genes with isoforms	−	11,044
Mean isoform number per gene	1	1.2
Genes with GO terms	16,569	17,265
Genes with a functional annotation	73,916	76,925
Complete BUSCOs	96.88%	97.85%
Complete and single-copy BUSCOs	8.75%	4.80%
Complete and duplicate BUSCOs	88.13%	93.05%
Fragmented BUSCOs	1.25%	0.90%
Missing BUSCOs	1.87%	1.25%

### Evaluation of the v1.0.a2 annotation

MAKER2^23^ was used to measure the consistency of gene loci with the available protein and nucleotide sequence alignments, the process of which was based on the mRNA quality index (QI) and annotation edit distance (AED). Each gene was assigned a QI score between 0 and 1, and a higher QI score suggests a higher proportion of exons that match the transcript alignment. The AED score is also between 0 and 1, where 0 indicates complete consistency with the evidence and where 1 means complete inconsistency with the evidence. The AED distribution is shifted toward lower (better) scores in v1.0.a2 compared with v1.0.a1 (Fig. 2b). In contrast, the cumulative QI distribution shows that the QI is shifted toward higher (better) scores in v1.0.a2 compared with v1.0.a1 (Fig. 2c). Therefore, v1.0.a2 has a higher proportion of gene models supported by the transcript evidence.

We used BUSCO v3.0.2^24^ (embryophyta_odb9 database) to assess the completeness of the v1.0.a1 and v1.0.a2 annotations. BUSCO measures genome assembly and annotation completeness based on a curated set of Plantae lineage-specific single-copy orthologs. With respect to the 1440 conserved genes, v1.0.a2 harbors 97.85% complete BUSCOs, compared to 96.88% harbored by v1.0.a1 (Table 1).

### Prediction of gene functions

To update the functional annotations of protein-coding genes in v1.0.a2, each of the predicted protein sequences was searched against the InterPro protein database via InterProScan^25^. Then, eggNOG mapper^26^ and PANNZER^27^ were employed to assign GO categories and KEGG functional annotations for all annotated loci. According to the eggNOG results, 17,265 genes were assigned to a specific GO term, compared to 16,569 genes in v1.0.a1 (Tables 1, S4). In addition, the iTAK pipeline^28^ was used to detect and classify transcription factors and protein kinases. A total of 5695 transcription factors (TFs) and 1340 transcriptional regulators (TRs) were identified in the v1.0.a2 annotation (Table S5). There were 170 more TF genes in v1.0.a2 than in v1.0.a1. Some transcription factor families acquired more members in v1.0.a2, such as the far-red impaired response 1 (FAR1) family, whose members increased from 41 to 163, the B3 family, whose members increased from 223 to 232, the Trihelix family, whose members increased from 108 to 115, and the MYB family, whose members increased from 414 to 440. Furthermore, there are 3611 protein kinase-encoding genes in v1.0.a2, 20 fewer than the number in v1.0.a1 (Table S5).

**Fig. 3:**
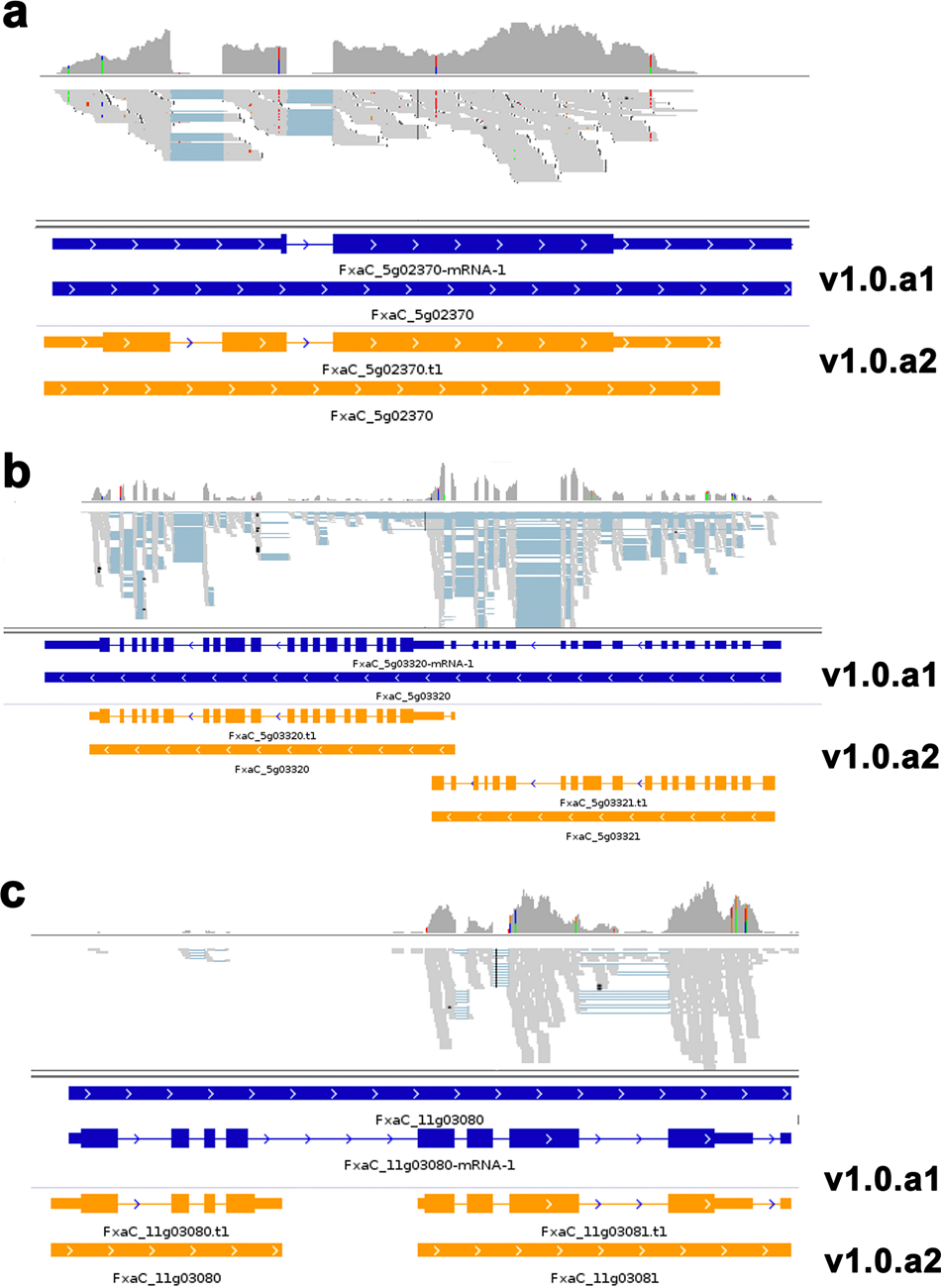
Examples of known genes with improved annotation. **a** IGV view of the gene model of FxaC_5g02370 according to the old (blue) and new (yellow) annotations. The blue and yellow lines with gene IDs below represent the whole gene. The blue and yellow lines with mRNA IDs below show the gene structure in detail. The thin bars represent UTRs, the thick bars represent ORFs, and the thin lines represent introns. The arrowheads within the bars indicate transcriptional orientation. **b** IGV view of the RNA-seq mapped reads for the two adjacent *FaAPM* genes (FxaC_5g03320 and FxaC_5g03321) in v1.0.a2 that correspond to a single gene in v1.0.a1. **c** FxaC_11g03080 in v1.0.a1 is split into two genes, FxaC_11g03080 and FxaC_11g03081

Below are several examples illustrating the improved accuracy of the v1.0.a2 annotation. FxaC_5g02370, a homolog of *AtMYB68* (AT5g65790) encoding an R2R3-MYB family protein with roles in root development^29^, has a new translation start site, resulting in a longer ORF (Fig. 3a). Two adjacent *FaAPM* genes (FxaC_5g03320 and FxaC_5g03321), both of which encode a homolog of AtAPM1 (AT4G33090) that interacts with secreted cell surface and cell wall proline-rich proteins^30^, were originally annotated as a single gene, with FxaC_5g03321 annotated as the 5′UTR of FxaC_5g03320, in v1.0.a1 (Fig. 3b). In addition, the single FxaC_11g03080 gene in v1.0.a1 is split into two genes (FxaC_11g03080 and FxaC_11g03081) in v1.0.a2; gene FxaC_11g03081 encodes a homolog of *AtTOD1* (AT5G46220) acting in turgor pressure regulation in both guard cells and pollen tubes in *Arabidopsis*^31^ (Fig. 3c).

### Reanalysis of the *F. ananassa* fruit transcriptomes

**Fig. 4:**
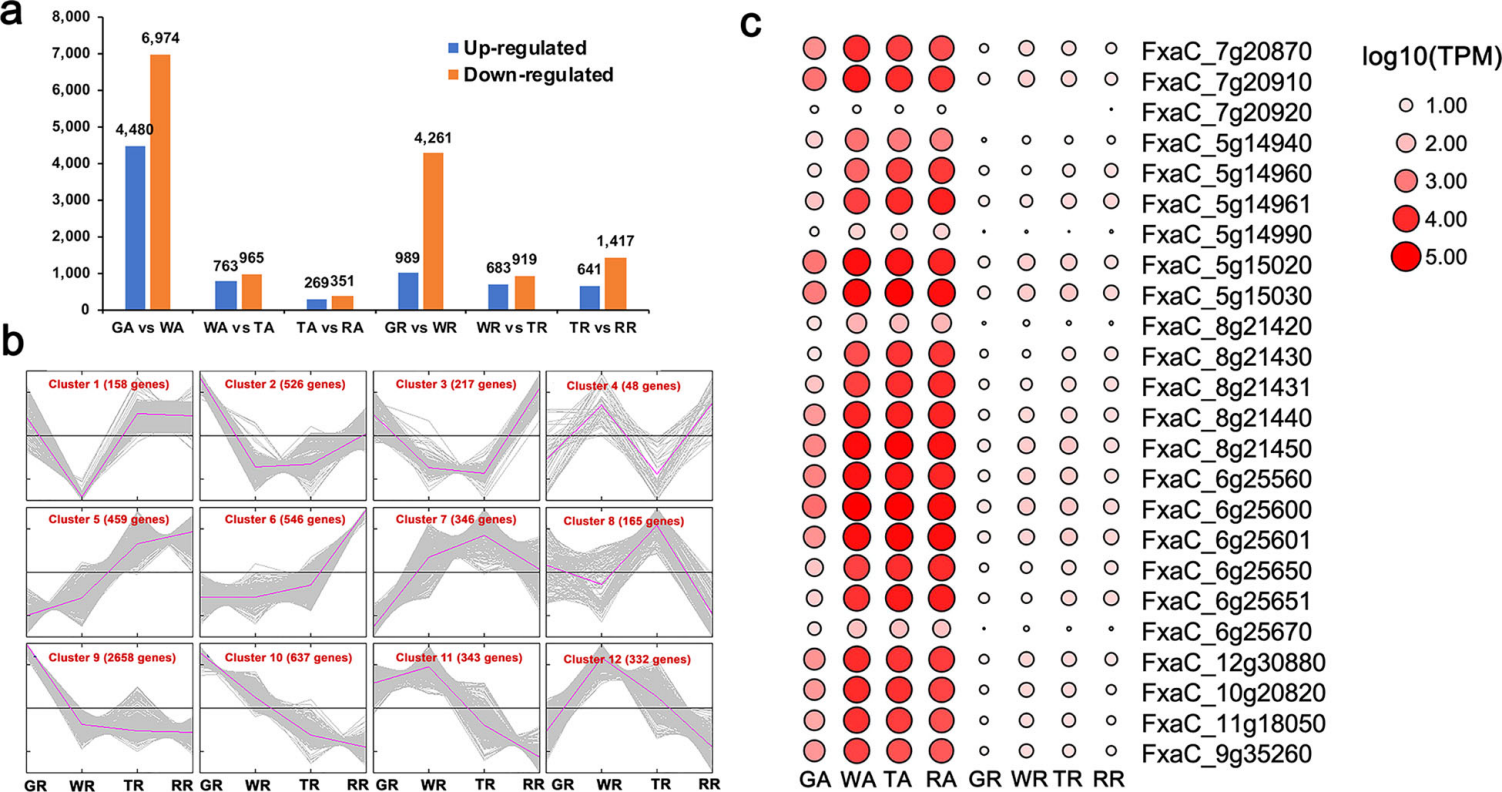
Differentially expressed genes (DEGs) during fruit development. **a** Number of differentially expressed genes identified in the successive transition from the green (G), white (W), turning (T), and red (R) stages in the receptacles (Rs) and in achene (As). **b** Average profiles of twelve gene clusters of genes in the receptacle. Line plot showing the expression of genes in the twelve gene clusters. The number of genes in each cluster is shown. The *Y*-axis represents the z-score obtained from the expression level (TPM). **c** Heatmap of *F. ananassa CRU1* genes expressed in different tissues. GR Green receptacle, WR White receptacle, TR Turning receptacle, RR Red receptacle, GA Green achene, WA White achene, TA Turning achene, RA Red achene

Several RNA-seq analyses of *F. ananassa* fruit development have been reported over the past several years^16,17^; these analyses have used the *F. vesca* genome as a reference genome due to the absence of a high-quality *F. ananassa* genome. In this study, we reanalyzed the transcriptome of the four stages of achenes and receptacles, the green (G), white (W), turning (T), and red (R) stages, with three biological replicates each^16^. Accurate gene expression profiling based on the new genome assembly and annotation should be helpful for genetic and functional research of strawberry fruit development and serves as a valuable resource. The gene read counts and expression profiles (transcripts per million (TPM)) of all the annotated genes in the achene and receptacle tissues across the four stages are presented in Table S6. A total of 42,624 genes (39.3% out of the 108,447 genes) were expressed at a level higher than 2 TPM in at least one of these tissues. The table also indicates their homologous genes in *Arabidopsis* and the corresponding gene annotation if available. In addition, we analyzed the global gene expression changes in the achenes and receptacles among the different stages, with the expression of more genes being downregulated than upregulated as ripening progressed (Fig. 4a, Tables S7, S8). In both the achenes and receptacles, the greatest number of DEGs between two consecutive stages was found between the green and white stages, which is consistent with the results of previous studies^16^. For receptacles, the top three enriched GO terms were alpha-amino acid catabolic process, cellular amino acid catabolic process, and carboxylic acid metabolic process, which are related to active metabolic processes occurring during fruit development.

To identify the transcriptional dynamics associated with fruit ripening, the DEGs with different expression profiles were assigned to different gene clusters using the K-means clustering approach. For the receptacle, a total of 6435 genes were found to be differentially expressed (padj < 0.01, fold change >2). All the DEGs were assigned to 12 coexpression clusters (Fig. 4b, Table S9). The expression pattern of cluster 10 exhibited a gradual decrease during receptacle development; this cluster was overrepresented with genes with GO terms associated with photosynthesis, generation of precursor metabolites, and energy-producing molecules (Table S10). Cluster 6, which consists of genes abundantly expressed in the red stage receptacle, was characterized by an abundance of genes associated with GO terms associated with catalytic activity. Many fruit ripening-related genes were in cluster 6. For example, *FaMYB10* (FxaC_2g30690), predominantly expressed in the red receptacle (RR), is a master transcriptional regulator of genes acting in the flavonoid/phenylpropanoid pathway during fruit ripening^32,33^. Finally, we clustered the genes differentially expressed during achene development (Fig. S1, Tables S11, S12). From green achenes (GAs) to white achenes (WAs), the expression levels of 4480 genes were significantly upregulated in WAs. Among these DEGs are 23 *CRU1* genes, homologs of *AtCRU1* (AT5G44120), which code for a 12S seed storage protein whose phosphorylation state is modulated in response to ABA in *Arabidopsis thaliana* seeds^34,35^. However, only 25 *CRU1* genes exist in *F. ananassa*, indicating that most *CRU1* genes are highly expressed in achenes after the white stage (Fig. 4c). This indicates that achenes, which contain seeds, accumulate storage proteins after the white stage.

### Strawberry genome database (SGD) based on the ViroBLAST webserver

**Fig. 5:**
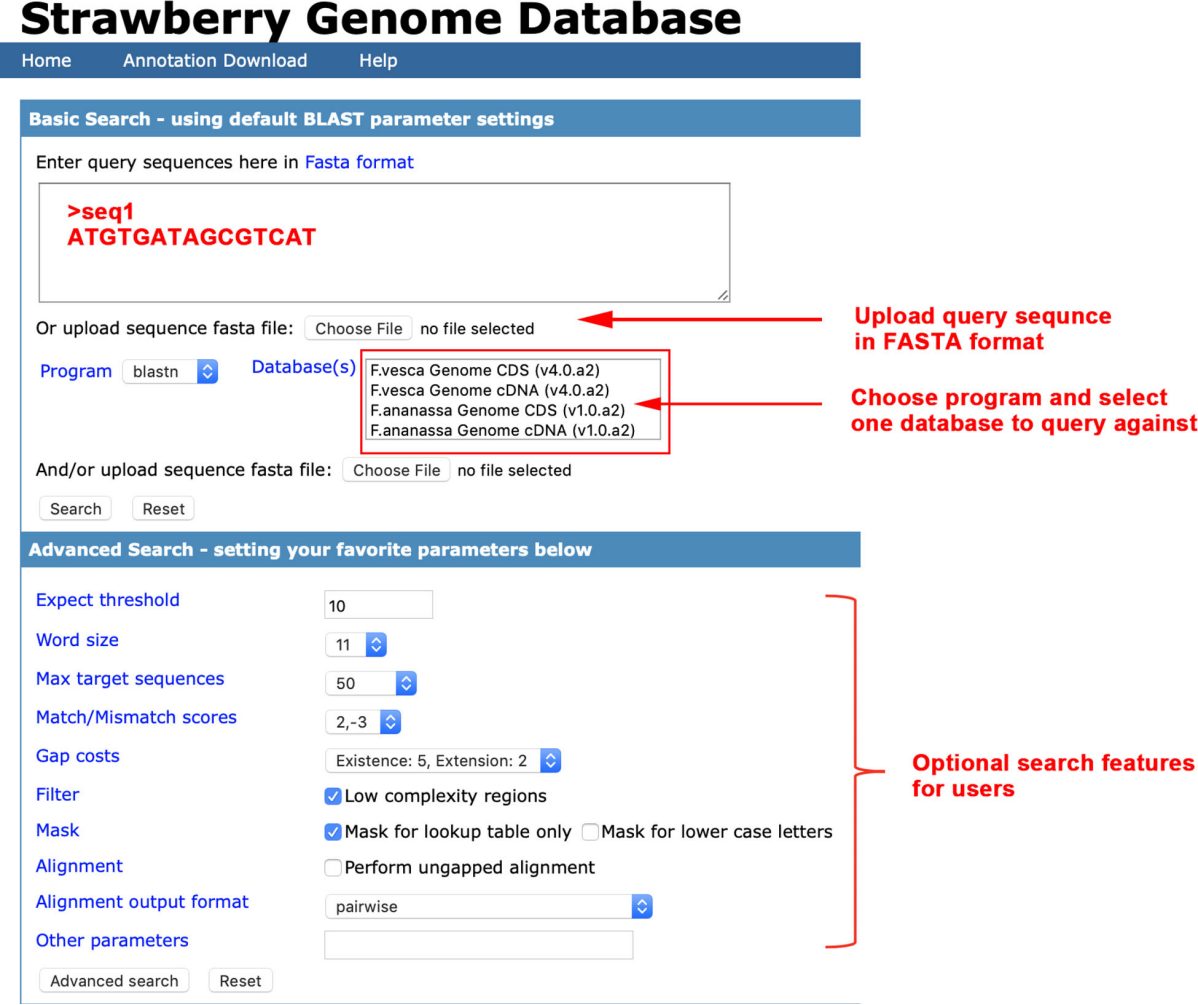
Image of the strawberry genome database website. Users can perform (batch) sequence searches against *F. vesca* and *F. ananassa* nucleotide and protein databases and can download annotation files. Users can also configure the advanced BLAST parameters

We constructed a SGD website for our new annotations (both *F. ananassa* (v1.0.a2) and *F. vesca* (v4.0.a2)), providing easy access to the *F. ananassa* and *F. vesca* genomes through downloading and homolog searching (http://www.strawberryblast.ml:8080/strawberry/viroblast.php) (Fig. 5). The SGD website, which is based on the ViroBLAST webserver^36^, allows users to search (via BLAST) the cDNA and protein sequences of both the *F. vesca* and *F. ananassa* genomes. Users can upload sequences as FASTA files or paste them into the query box directly. Additionally, the website provides more BLAST options for advanced users to obtain more specific information.

## Conclusion

Recently, a near-complete chromosome-scale assembly of cultivated strawberry (*F. ananassa*) has become available^2^. An annotation (v1.0.a1) based on 16 Illumina RNA-Seq datasets generated from different samples accompanied this *F. ananassa* genome. However, the short Illumina reads from a small number and type of tissues limited the gene annotations. Here, we used an optimized annotation pipeline to improve the *F. ananassa* genome annotation by taking advantage of PacBio full-length transcripts from mixed tissues and Illumina short reads derived from 92 different libraries (Table S1). In this new v1.0.a2 annotation, a total of 360 new genes were identified, and 19,174 gene models were modified and improved. Some transcription factor families changed dramatically in the number of their member genes. For instance, the number of *far-red impaired response 1* (*FAR1*) genes increased from 41 to 163. We also added transcript isoforms and 5′UTR and 3′UTR information to the new annotation. Furthermore, we reanalyzed previous RNA-seq data^16^ from receptacles and achenes at the four ripening stages of *F. ananassa* and found that achenes begin to accumulate storage proteins soon after the white stage, when the fruit begins to ripen. In addition, by requantifying previous RNA-seq data based on alignment to the newly annotated genome, we provide expression profiles of all the genes in the newly annotated genome across multiple fruit tissues and stages. Together, the updated annotation and gene expression quantification during fruit development will serve as valuable resources for gene functional studies in cultivated strawberry and for genome comparisons in the *Rosaceae* family.

## Materials and methods

### Transcriptome datasets used in this study

Publicly available SMRT and Illumina data (Table S1) were downloaded to reannotate the genome (v1.0, 10.5061/dryad.b2c58pc)^2^. A SMRT library was generated from RNAs isolated from six pooled tissues, including dwarf stem, flower, mature leaf, and fruit tissues^12^. Ninety-two Illumina-based RNA-seq datasets were obtained from flower, petal, leaf, root, stem, and fruit samples from in *F. ananassa* at different developmental stages or under different treatments, including ABA and NDGA5^2,14–18^.

### Read processing

The first 12 bp of the Illumina RNA-Seq reads with a phred quality <= Q15 were trimmed using fastp software v0.20.0^37^. The clean reads for each library were then aligned individually to the *F. ananassa* genome^2^ using STAR (v2.7.2b)^38^. Only the uniquely mapped reads were retained for downstream analysis. In addition, the SMRT analysis software suite (v2.3.0) was used to analyze the PacBio SMRT sequencing data; reads with a quality score >90 and full pass number >2 were retained. The LoRDEC toolkit v0.9^39^ was employed to further correct the SMRT full-length transcripts using Illumina short reads, with the following parameters: -s 3 -k 19.

### Comprehensive transcriptome generation

The aligned short reads were assembled into transcripts for each library using StringTie v2.0^40^; the default parameters were used, except that the minimum isoform fraction was set to 0.2 to remove transcripts with low expression. The de novo assembly of all the RNA-seq reads was performed by Trinity v2.5.1^41^ with the default settings. The corrected SMRT transcripts were then aligned to the *F. ananassa* genome by GMAP version 2015-07-23^42^, with >90% alignment identity and >85% alignment coverage. The genome-guided assembly, de novo assembly, and aligned PacBio data were then integrated into a comprehensive transcriptome that was used to construct the best gene models by PASA v2.4.1^43^.

### Annotation of the *F. ananassa* genome

A soft-masked genome was generated by RepeatMasker v4.1.0^44^ using the repeats identified by RepeatModeler. BRAKER v2.1.4 was employed to generate initial gene models based on the different types of evidence^19^. BRAKER was run with the following evidence: 1) intron hints converted from mapped short Illumina reads; 2) trained models from BRAKER with full-length transcripts; 3) intron hints converted from SMRT full-length transcripts; 4) protein hints converted from *F. vesca* v4.0.a2 proteins, *Arabidopsis* proteins, and UniProt proteins mapped to the genome by Exonerate v2.2.0; and 5) the *F. ananassa* genome repeat masked.

EVidenceModeler (EVM) v1.1.1^20^ identified the consensus gene models among the BRAKER gene models, *F. ananassa* v1.0.a1 gene models, SMRT full-length transcripts, genome-guided transcripts from Illumina RNA-Seq, de novo transcripts from Illumina RNA-Seq, UniProt proteins, *Arabidopsis* proteins, and *F. vesca* v4.0.a2 proteins with a nonstochastic weighted value; the weight value for each line of evidence was set to 8, 9, 12, 10, 6, 3, 4, and 8, respectively, according to the accuracy of the evidence (checked in IGV). PASA v2.4.1^43^ refined the gene models by adding alternatively spliced isoforms, adding UTR annotations, and modifying gene structures. Additionally, the genome annotation was manually curated using Apollo^45^ on the basis of RNA-seq alignment from different RNA-seq libraries.

### Differential expression analysis during achene and receptacle development

The raw gene read counts were measured by featureCounts (-t CDS -g Parent -p)^46^. TPM was used to represent the gene expression level. Differential expression analysis was performed by the R package DESeq2^47^ using the raw counts. Genes with a padj <0.01 and a fold change >2 were considered differentially expressed genes. GO enrichment analysis was conducted by AgriGO v2.0^48^ using a hypergeometric distribution. We used the Yekutieli (FDR under dependency) multitest adjustment method to correct for the *P*-value, and the FDR cutoff was set to 0.05. Finally, all the differentially expressed genes among equivalent tissues across stages were subjected to K-means clustering with the euclidean distance metric in MeV 4.8.1^49^. The *Z*-score was calculated to reflect the expression level of genes in each cluster. The heatmap was produced by TBtools v1.055^50^.

## Supplementary information

Figure S1

Dataset 1

Dataset 2

Dataset 3

Dataset 4

Dataset 5

Dataset 6

Dataset 7

Dataset 8

Dataset 9

Dataset 10

Dataset 11

Dataset 12

## Data Availability

Table S2 is the gff3 file of annotation v1.0.a2, which is also available through our strawberry genome database website (http://www.strawberryblast.ml:8080/strawberry/docs/download.html) and the GDR (https://www.rosaceae.org/).

## Supplementary information

**Supplementary information** The online version contains supplementary material available at 10.1038/s41438-021-00476-4.
