# Supplementary figures and images for: Reannotation of the cultivated strawberry genome and establishment of a strawberry genome database
Source: Hortic Res. 2021 Mar 1;8:41. doi: 10.1038/s41438-021-00476-4 (PMC7917095; doi:10.1038/s41438-021-00476-4)

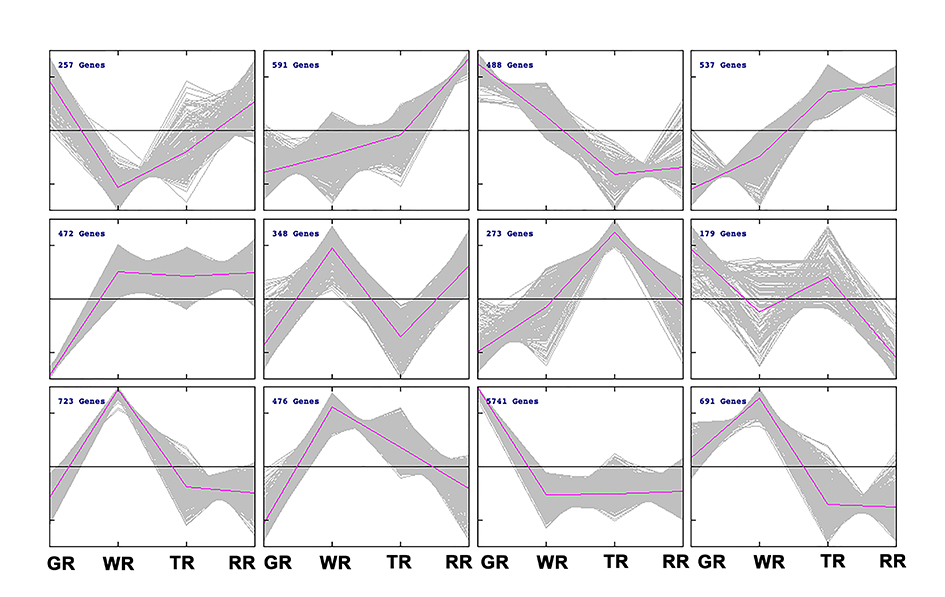

Supplement: Supplementary file 1 — Figure S1 [file 41438_2021_476_MOESM1_ESM.jpg]
